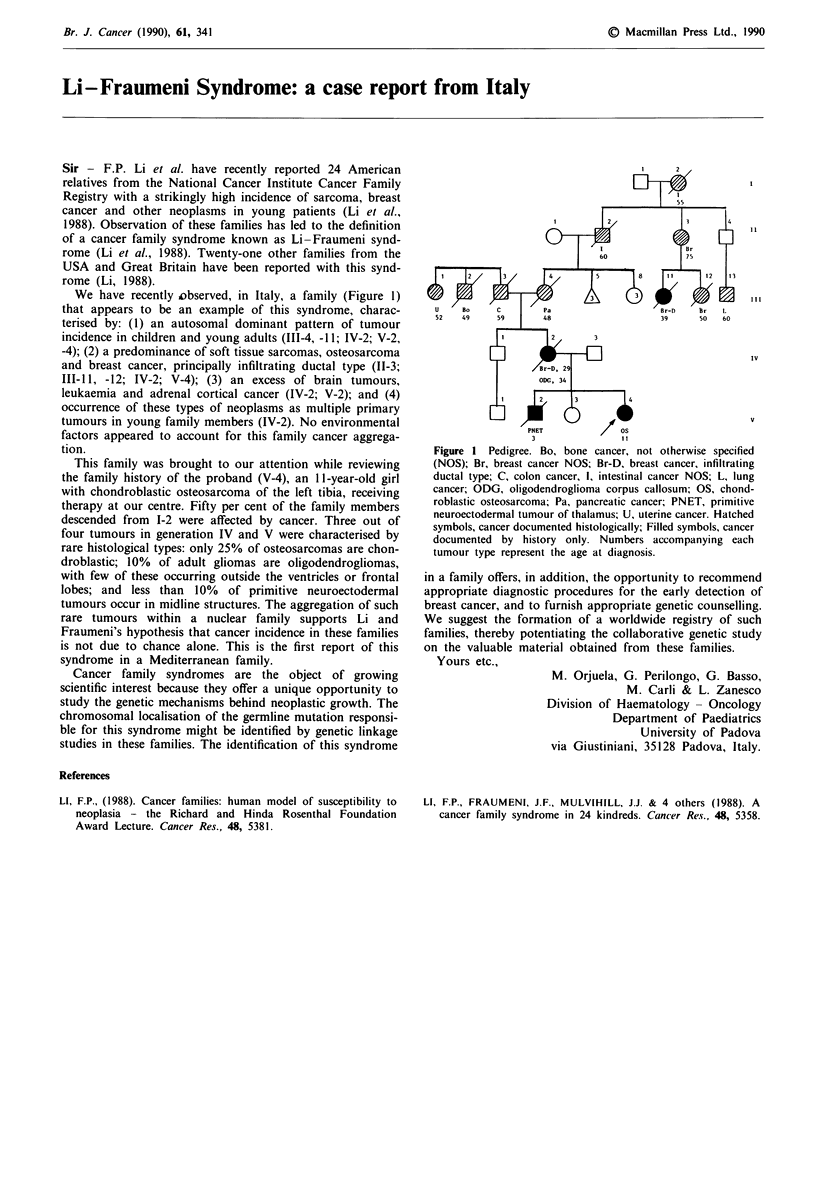# Li-Fraumeni syndrome: a case report from Italy.

**DOI:** 10.1038/bjc.1990.70

**Published:** 1990-02

**Authors:** M. Orjuela, G. Perilongo, G. Basso, M. Carli, L. Zanesco


*Br. J. Cancer* (1990), **61**, 341
© Macmillan Press Ltd., 1990

### Li–Fraumeni Syndrome: a case report from Italy

**Sir** – F.P. Li *et al.* have recently reported 24 American relatives from the National Cancer Institute Cancer Family Registry with a strikingly high incidence of sarcoma, breast cancer and other neoplasms in young patients (Li *et al.*, 1988). Observation of these families has led to the definition of a cancer family syndrome known as Li–Fraumeni syndrome (Li *et al.*, 1988). Twenty-one other families from the USA and Great Britain have been reported with this syndrome (Li, 1988).

We have recently observed, in Italy, a family (Figure 1) that appears to be an example of this syndrome, characterised by: (1) an autosomal dominant pattern of tumour incidence in children and young adults (III-4, -11; IV-2; V-2, -4); (2) a predominance of soft tissue sarcomas, osteosarcoma and breast cancer, principally infiltrating ductal type (II-3; III-11, -12; IV-2; V-4); (3) an excess of brain tumours, leukaemia and adrenal cortical cancer (IV-2; V-2); and (4) occurrence of these types of neoplasms as multiple primary tumours in young family members (IV-2). No environmental factors appeared to account for this family cancer aggregation.

This family was brought to our attention while reviewing the family history of the proband (V-4), an 11-year-old girl with chondroblastic osteosarcoma of the left tibia, receiving therapy at our centre. Fifty per cent of the family members descended from I-2 were affected by cancer. Three out of four tumours in generation IV and V were characterised by rare histological types: only 25% of osteosarcomas are chondroblastic; 10% of adult gliomas are oligodendrogliomas, with few of these occurring outside the ventricles or frontal lobes; and less than 10% of primitive neuroectodermal tumours occur in midline structures. The aggregation of such rare tumours within a nuclear family supports Li and Fraumeni's hypothesis that cancer incidence in these families is not due to chance alone. This is the first report of this syndrome in a Mediterranean family.

Cancer family syndromes are the object of growing scientific interest because they offer a unique opportunity to study the genetic mechanisms behind neoplastic growth. The chromosomal localisation of the germline mutation responsible for this syndrome might be identified by genetic linkage studies in these families. The identification of this syndrome in a family offers, in addition, the opportunity to recommend appropriate diagnostic procedures for the early detection of breast cancer, and to furnish appropriate genetic counselling. We suggest the formation of a worldwide registry of such families, thereby potentiating the collaborative genetic study on the valuable material obtained from these families.

**Figure 1** Pedigree. Bo, bone cancer, not otherwise specified (NOS); Br, breast cancer NOS; Br-D, breast cancer, infiltrating ductal type; C, colon cancer, I, intestinal cancer NOS; L, lung cancer; ODG, oligodendroglioma corpus callosum; OS, chondroblastic osteosarcoma; Pa, pancreatic cancer; PNET, primitive neuroectodermal tumour of thalamus; U, uterine cancer. Hatched symbols, cancer documented histologically; Filled symbols, cancer documented by history only. Numbers accompanying each tumour type represent the age at diagnosis.

Yours etc.,

M. Orjuela, G. Perilongo, G. Basso,
M. Carli & L. Zanesco
Division of Haematology – Oncology
Department of Paediatrics
University of Padova
via Giustiniani, 35128 Padova, Italy.

**References**

LI, F.P., (1988). Cancer families: human model of susceptibility to neoplasia – the Richard and Hinda Rosenthal Foundation Award Lecture. *Cancer Res.*, **48**, 5381.

LI, F.P., FRAUMENI, J.F., MULVIHILL, J.J. & 4 others (1988). A cancer family syndrome in 24 kindreds. *Cancer Res.*, **48**, 5358.